# Small intestinal gastrointestinal stromal tumour with a coexisting retroperitoneal tumour: a case report and institutional review

**DOI:** 10.1093/jscr/rjag667

**Published:** 2026-07-31

**Authors:** Hana Futagami, Yusuke Yoshida, Ryohei Shoji, Yuki Matsumi

**Affiliations:** Department of Gastroenterological Surgery, Okayama University Graduate School of Medicine, Dentistry and Pharmaceutical Sciences, Okayama, Japan; Department of Gastroenterological Surgery, Okayama University Graduate School of Medicine, Dentistry and Pharmaceutical Sciences, Okayama, Japan; Department of Surgery, Okayama Saiseikai General Hospital, Okayama, Japan; Department of Gastroenterological Surgery, Okayama University Graduate School of Medicine, Dentistry and Pharmaceutical Sciences, Okayama, Japan; Department of Gastroenterological Surgery, Okayama University Graduate School of Medicine, Dentistry and Pharmaceutical Sciences, Okayama, Japan

**Keywords:** small intestinal GIST, retroperitoneal GITS, laparoscopy, sporadic

## Abstract

Gastrointestinal stromal tumours (GISTs) most commonly arise in the stomach or small intestine, whereas retroperitoneal lesions are rare. A 69-year-old woman without neurofibromatosis type 1 underwent single-incision laparoscopic resection for a high-risk jejunal GIST. Four months later, follow-up computed tomography revealed a 25-mm retroperitoneal tumour adjacent to the right common iliac artery, which had been retrospectively identifiable on the initial imaging. Laparoscopic excision was successfully performed, and pathological examination confirmed GIST. The patient resumed adjuvant imatinib therapy and remains disease-free at 12 months. Retrospective review of 18 surgically treated small intestinal GISTs at our institution showed that most multiple lesions were associated with neurofibromatosis type 1 or peritoneal dissemination, whereas the present case represented an exceptionally rare sporadic presentation. Careful imaging assessment and postoperative surveillance are essential for detecting atypical extra-gastrointestinal lesions.

## Introduction

Gastrointestinal stromal tumours (GISTs) are the most common mesenchymal tumours of the gastrointestinal tract, thought to originate from interstitial cells of Cajal. They predominantly arise in the stomach (40%–60%) or small intestine (20%–30%) [1–5]. Small intestinal GISTs tend to be larger than gastric GISTs and are often diagnosed at advanced stages due to delayed symptom onset [5, 6].

Multiple GISTs are extremely rare in sporadic cases and are typically associated with hereditary syndromes, particularly neurofibromatosis type 1 (NF1) [7–9]. Retroperitoneal GISTs account for <5%–10% of all GISTs. Although the exact pathogenesis remains debated, most retroperitoneal GISTs represent exophytic growth from a gastrointestinal origin, and true primary extra-gastrointestinal GISTs are exceedingly rare [10–12].

Herein, we report a rare case of small intestinal GIST associated with a retroperitoneal lesion and present a retrospective review of surgically treated small intestinal GISTs at our institution.

## Case presentation

A 69-year-old woman presented with fatigue and was referred for further evaluation of anemia (haemoglobin 10 g/dl). She had no clinical signs of NF1 and no family history of GIST.

Endoscopy revealed a submucosal ulcerated lesion in the proximal jejunum, ~15 cm distal to the ligament of Treitz (Fig. 1). Contrast-enhanced computed tomography (CT) showed an exophytic mass in the proximal jejunum (Fig. 2a). Due to active bleeding, biopsy was deferred, and diagnostic and therapeutic single-incision laparoscopic jejunal resection was performed (Fig. 3). The operation lasted 98 minutes with minimal blood loss, and postoperative recovery was uneventful. The patient was discharged on postoperative day 9.

**Figure 1:**
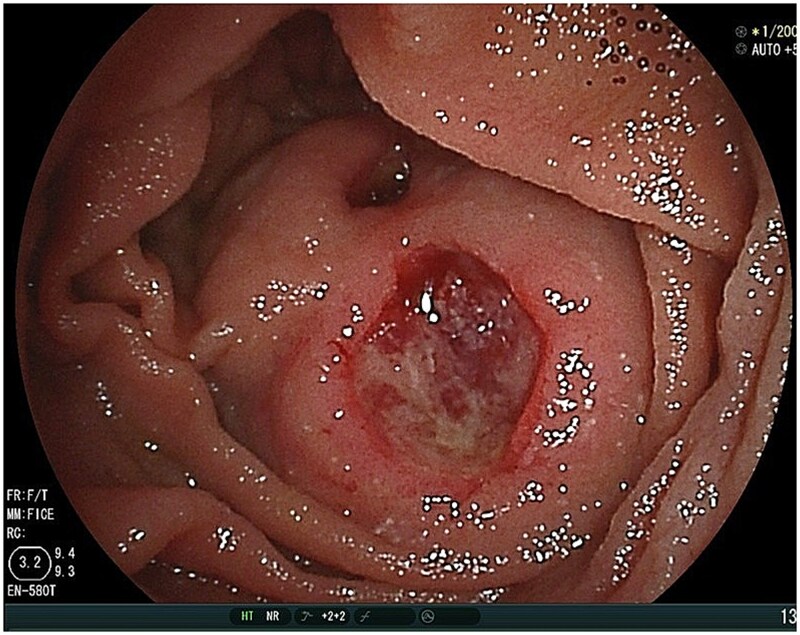
Small intestinal endoscopy. A submucosal tumour-like elevated lesion with ulceration is observed in the jejunum, ~15 cm distal to the ligament of Treitz.

**Figure 2:**
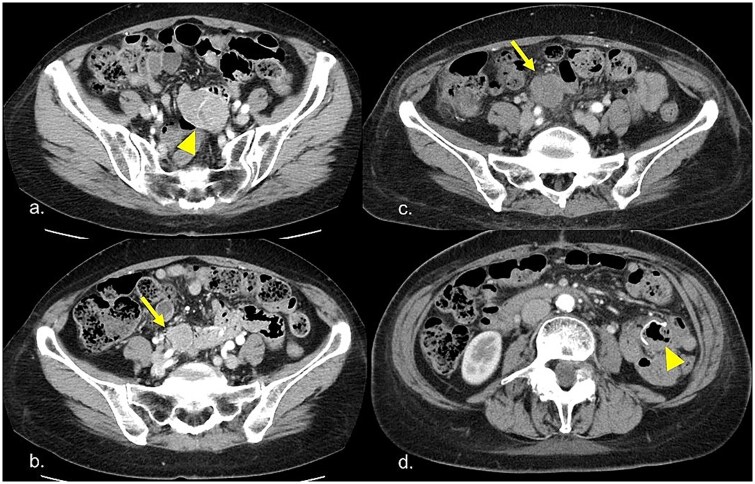
Abdominal CT findings. (a) Preoperative CT before the initial surgery showing an exophytic enhancing mass in the proximal jejunum (arrow head). (b) Retrospective review of the initial CT reveals a mass adjacent to the ventral side of the right common iliac artery (arrow). (c) CT at 4 months postoperatively demonstrating the mass adjacent to the ventral side of the right common iliac artery (arrow). (d) CT at 4 months postoperatively showing the anastomosis from the initial surgery (arrow head).

**Figure 3:**
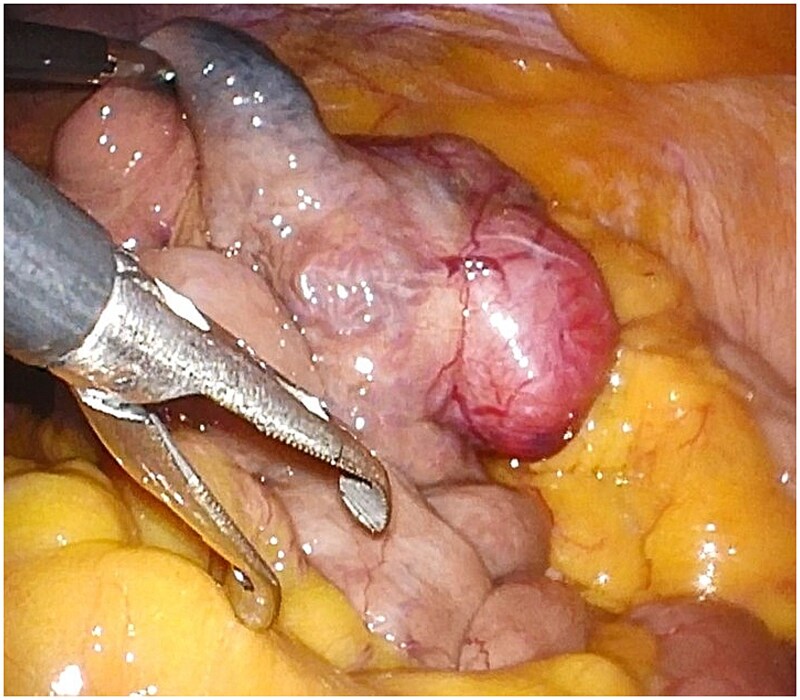
Intraoperative photograph during the initial surgery. An exophytic mass is observed in the proximal jejunum.

Histopathology demonstrated spindle cell proliferation, and immunohistochemistry was positive for c-kit and Discovered on GIST-1 (DOG1), consistent with a jejunal GIST (Fig. 4a–e). According to the modified Fletcher classification, the tumour was high-risk (tumour size: 5.5 cm, mitotic index: 3 per 5 mm^2^). Imatinib therapy was initiated 1 month postoperatively.

**Figure 4:**
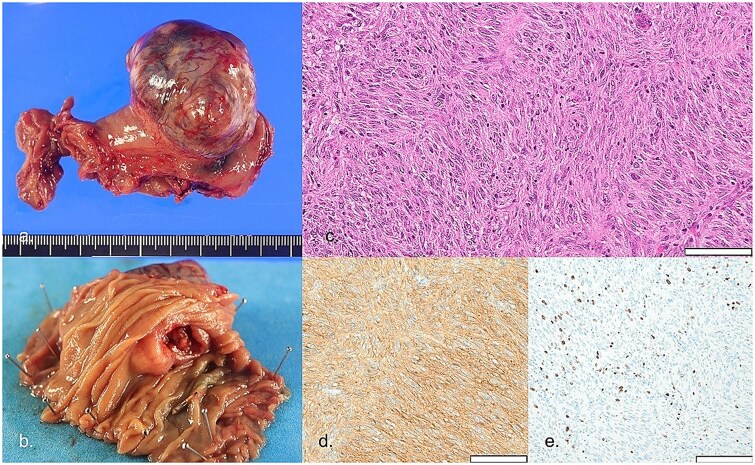
Resected specimen and histopathological findings from the initial surgery. (a, b) Macroscopic appearance of the resected small intestinal tumour. (c) Haematoxylin and eosin staining showing spindle cell proliferation (×200). (d) Immunohistochemical staining positive for c-kit (CD117) (×200). (e) Ki-67 staining demonstrating the proliferative activity (×200). White bar: 100 μm.

At 4 months follow-up, CT revealed a 25 mm retroperitoneal mass adjacent to the right common iliac artery (Fig. 2c). The lesion was distant from the anastomotic site created during the initial surgery (Fig. 2d). The lesion was noted retrospectively on preoperative CT (Fig. 2b). Laparoscopic excision was performed; there was no evidence of hepatic metastasis or peritoneal dissemination. The tumour was well-encapsulated and safely resected (Fig. 5a–d). Pathology confirmed a retroperitoneal GIST (Supplementary Fig. 1a–d). Postoperatively, imatinib was resumed, and the patient remains disease-free at 12 months of follow-up.

**Figure 5:**
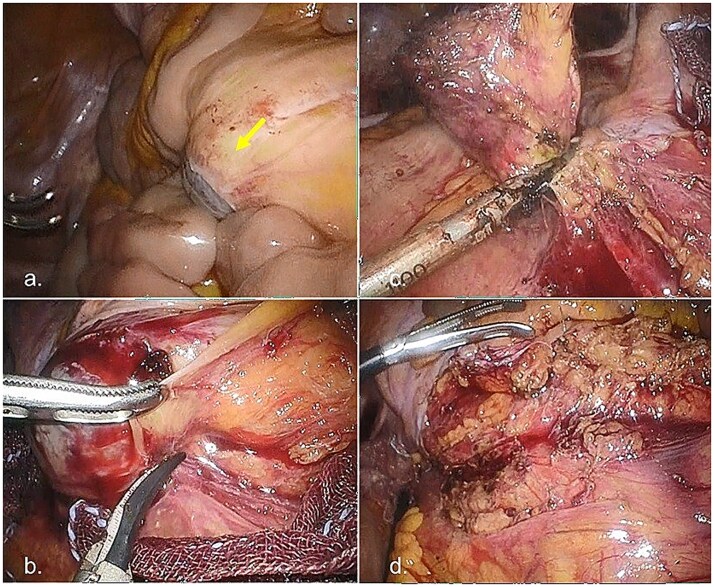
Intraoperative photographs during the second surgery. (a) The tumor is observed near the right iliac angle (arrow). (b) Dissection was started from the cranial side of the tumor toward the dorsal aspect. (c) The tumour was completely excised without breaching the capsule. (d) The operative field after tumour removal.

## Institutional case series

Between January 2015 and December 2025, 18 patients with primary small intestinal GISTs (excluding duodenal tumours) underwent surgical resection at our institution. Tumours were solitary in 12 patients and multiple in 6 patients (33%) (Table 1). Two of the latter cases represented peritoneal dissemination. Among the remaining four patients with non-disseminated multiple tumours, three were associated with NF1 and involved multiple small intestinal GISTs. In the present case, both tumours were encapsulated.

**Table 1:** Clinical characteristics of six patients with multiple small intestinal GISTs

Case	Age	Sex	No. of primary tumors	Location	NF1	Peritoneal dissemination	Retroperitoneal lesion
1	87	F	Single	Jejunum	No	Yes	No
2	54	M	Multiple	Jejunum and ileum	Yes	No	No
3	68	M	Single	Jejunum	No	Yes	No
4	76	M	Multiple	Jejunum and ileum	Yes	No	No
**5**	**69**	**F**	—	**Jejunum**	**No**	**No**	**Yes**
6	53	F	Multiple	Jejunum and ileum	Yes	No	No

NF1, neurofibromatosis type 1.

## Discussion

Small intestinal GISTs are often larger than gastric GISTs and may remain asymptomatic until advanced stages [5, 6].

Sporadic multiple or synchronous GISTs are exceedingly rare; most multiple lesions are associated with NF1 or hereditary syndromes [7–9]. In our series, although six patients had multiple lesions, only one (the present case) had a sporadic small intestinal GIST associated with a retroperitoneal lesion in the absence of NF1. Although genetic analysis was not performed for our patient, the absence of NF1 features or family history suggests a sporadic case.

A critical issue in the present case is whether the retroperitoneal lesion represents a true primary tumour or metastasis from the small intestinal GIST. In general, metastatic GISTs commonly involve the liver or peritoneum and often present as multiple nodules with infiltrative features or variable tumour size [13, 14]. In contrast, the retroperitoneal tumour in this case was solitary, well-circumscribed, and encapsulated, without evidence of capsular disruption or invasive growth. These features are atypical for metastatic disease.

Furthermore, although extra-gastrointestinal GISTs (EGISTs) are defined as tumours arising in the retroperitoneum, mesentery, or omentum, it has been suggested that many so-called EGISTs actually represent exophytic growth from an unrecognized gastrointestinal primary or metastatic lesions [10]. Therefore, the distinction between true primary EGIST and metastasis remains challenging. In the present case, the absence of peritoneal dissemination, the solitary nature of the lesion, and its well-defined morphology are not typical of metastatic disease.

Although genetic analysis was not performed, which limits definitive conclusions, and histopathological comparison between the two lesions was limited because the retroperitoneal tumour was resected after initiation of imatinib therapy, which may induce treatment-related changes. These changes can obscure mitotic activity and proliferation indices, making direct comparison difficult. Therefore, the distinction between synchronous primary tumours and metastatic disease could not be definitively established based on pathological findings alone. Retroperitoneal GISTs are rare, and their pathogenesis is debated. Miettinen et al. reported that most retroperitoneal GISTs mimic exophytic gastrointestinal tumours, and true primary extra-gastrointestinal GISTs are extremely rare [10]. Resectable retroperitoneal GISTs can achieve prolonged survival despite generally poor prognosis for advanced cases. Li et al. suggested that sporadic multiple GISTs may occasionally involve retroperitoneal lesions [9]; however, to our knowledge, no previous report has described small intestinal and retroperitoneal GISTs without NF1.

Minimally invasive surgery proved both useful and limited in this setting: while laparoscopic-assisted access facilitated the identification of multiple intestinal lesions, detection of the retroperitoneal tumour was more difficult, although complete laparoscopic resection was ultimately achieved. Furthermore, this case emphasizes the importance of postoperative imaging and individualized treatment strategies, as early detection of the retroperitoneal lesion through follow-up imaging enabled safe and successful resection.

Because molecular analysis was not performed, the relationship between the two tumours remains speculative. Therefore, the present report should be interpreted as describing a rare clinicopathological presentation rather than providing definitive evidence of synchronous primary GISTs.

## Conclusion

We reported a rare case of small intestinal GIST associated with a retroperitoneal lesion. This case highlights the diagnostic challenges in distinguishing synchronous primary tumours from metastatic disease. It also underscores the importance of careful postoperative imaging follow-up and awareness of potential extra-gastrointestinal lesions. Clinicians should recognize that coexisting extra-gastrointestinal lesions may represent either atypical metastatic disease or an independent lesion, and careful clinicopathological evaluation is required.

## Supplementary Material

Supple_fig_rjag667

Supple_figure_1_rjag667

## Data Availability

The data that support the findings of this study are available from the corresponding author upon reasonable request.
