# Supplementary figures and images for: Small intestinal gastrointestinal stromal tumour with a coexisting retroperitoneal tumour: a case report and institutional review
Source: J Surg Case Rep. 2026 Jul 31;2026(7):rjag667. doi: 10.1093/jscr/rjag667 (PMC13426727; doi:10.1093/jscr/rjag667)

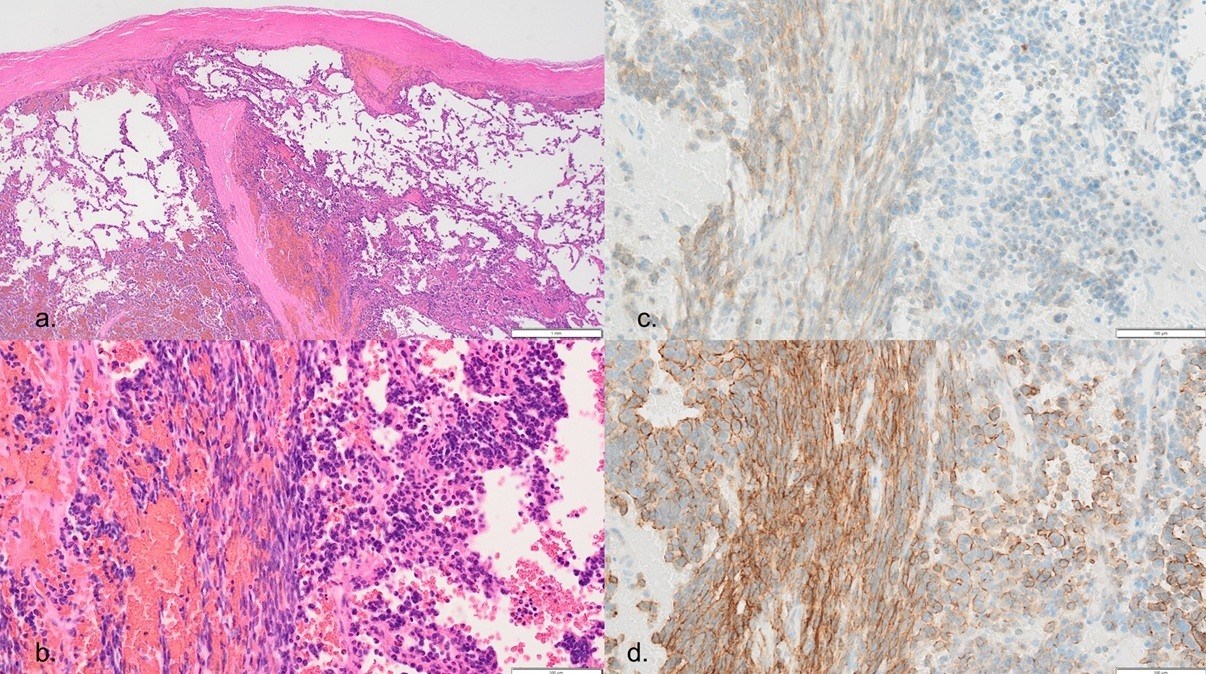

Supplement: Supple_fig_rjag667 [file supple_fig_rjag667.jpeg]
