# Supplementary material for: Small intestinal gastrointestinal stromal tumour with a coexisting retroperitoneal tumour: a case report and institutional review
Source: J Surg Case Rep. 2026 Jul 31;2026(7):rjag667. doi: 10.1093/jscr/rjag667 (PMC13426727; doi:10.1093/jscr/rjag667)
Supplement: Supple_figure_1_rjag667 [file supple_figure_1_rjag667.docx]

**Supple figure 1.** Histopathological findings of the retroperitoneal GIST.

a. Low-power view (×20) showing a well-encapsulated tumor with extensive intratumoral hemorrhage and necrosis, likely reflecting treatment effects.
b. High-power view (×200).
c. Immunohistochemical staining positive for c-kit (CD117) (×200).

d. Immunohistochemical staining positive for DOG1 (×200).
